# Promotion of Physical Activity in Older People Using mHealth and eHealth Technologies: Rapid Review of Reviews

**DOI:** 10.2196/22201

**Published:** 2020-12-29

**Authors:** Lisa McGarrigle, Chris Todd

**Affiliations:** 1 School of Health Sciences Faculty of Biology, Medicine and Health The University of Manchester Manchester United Kingdom; 2 Manchester Academic Health Science Centre Manchester United Kingdom; 3 National Institute for Health Research Older People and Frailty Policy Research Unit School of Health Sciences Faculty of Biology, Medicine and Health, The University of Manchester Manchester United Kingdom; 4 Manchester University NHS Foundation Trust Manchester United Kingdom; 5 National Institute for Health Research Applied Research Collaboration-Greater Manchester School of Health Sciences Faculty of Biology, Medicine and Health, The University of Manchester Manchester United Kingdom

**Keywords:** physical activity, mHealth, eHealth, app, accelerometer, pedometer, technology, COVID-19

## Abstract

**Background:**

Older people are at increased risk of adverse health events because of reduced physical activity. There is concern that activity levels are further reduced in the context of the COVID-19 pandemic, as many older people are practicing physical and social distancing to minimize transmission. Mobile health (mHealth) and eHealth technologies may offer a means by which older people can engage in physical activity while physically distancing.

**Objective:**

The objective of this study was to assess the evidence for mHealth or eHealth technology in the promotion of physical activity among older people aged 50 years or older.

**Methods:**

We conducted a rapid review of reviews using PRISMA (Preferred Reporting Items for Systematic Reviews and Meta-Analyses) guidelines. We searched for systematic reviews published in the English language in 3 electronic databases: MEDLINE, CINAHL Plus, and Scopus. Two reviewers used predefined inclusion criteria to select relevant reviews and extracted data on review characteristics and intervention effectiveness. Two independent raters assessed review quality using the AMSTAR-2 tool.

**Results:**

Titles and abstracts (n=472) were screened, and 14 full-text reviews were assessed for eligibility. Initially, we included 5 reviews but excluded 1 from the narrative as it was judged to be of critically low quality. Three reviews concluded that mHealth or eHealth interventions were effective in increasing physical activity. One review found that the evidence was inconclusive.

**Conclusions:**

There is low to moderate evidence that interventions delivered via mHealth or eHealth approaches may be effective in increasing physical activity in older adults in the short term. Components of successful interventions include self-monitoring, incorporation of theory and behavior change techniques, and social and professional support.

## Introduction

Older people engage in physical activity less regularly than younger age groups, and participation progressively decreases with age [1]. Inactivity is associated with frailty and adverse health outcomes in middle-aged to older adults [2], with sedentary people aged >50 years having twice the risk of death compared to those with the highest levels of physical activity [3]. The benefits of engagement in physical activity in older age are vast. Regular activity of moderate intensity (150 minutes per week) is consistently associated with reduced risk of chronic diseases [4], cognitive decline [5], and mortality [6]. Exercise programs that emphasize improving strength and balance reduce falls in older people [7].

During 2020, as a result of lockdown and physical or social distancing measures introduced in an effort to reduce coronavirus (COVID-19) transmission, there is concern that older people are at risk of further reduced activity levels and consequently, at increased risk of adverse health events. One approach to promoting activity that has gained particular traction over recent years is the use of exercise and activity mobile apps, tracking devices, and tablet or computer-based interventions. Physical activity interventions delivered via apps on mobile or wireless devices, such as smartphones or tablets, are collectively referred to as mobile health (mHealth) interventions [8]. Interventions delivered or enhanced through the internet and related technologies (eg, websites, wearable motion sensing devices) are referred to as eHealth interventions [9]. These kinds of interventions may offer a cost-effective and accessible way to promote activity in older populations as an alternative to face-to-face sessions. A recent review and meta-analysis of randomized controlled trials (RCTs) across a range of age groups found that mHealth interventions increased physical activity levels and reduced sedentary behavior [10]. mHealth and eHealth approaches may be feasible and acceptable in older populations [11], with an increasing number of older adults accessing the internet in recent years [12]. The effectiveness of these kinds of interventions in older people has been widely investigated, but a clear consensus on their usefulness in increasing physical activity is lacking. The objective of this review was to summarize the evidence from systematic reviews of the effectiveness of mHealth and eHealth approaches on physical activity in older people.

## Methods

To provide an overview the evidence on the use of mHealth and eHealth technologies in the promotion of physical activity in older people, we undertook a rapid review of reviews [13,14] and followed guidance for conducting overviews [15]. We followed a standard protocol in accordance with the PRISMA (Preferred Reporting Items for Systematic Reviews and Meta-analysis) statement, adjusted for rapid review and “review of reviews” methodologies.

### Search Strategy

Database searches were conducted in MEDLINE (Ovid), CINAHL Plus (EBSCO), and Scopus (Elsevier) for reviews focusing on mHealth or eHealth technologies in the promotion of physical activity in older people. All searches were conducted in May 2020 (see Multimedia Appendix 1 for the search strategy).

### Inclusion and Exclusion Criteria

As this was a rapid review, included papers were restricted to full-text availability in the English language. No restrictions were made for year of publication nor for study designs included in the reviews in order to get a complete picture of the effectiveness of the interventions.

Reviews were included if they used systematic review techniques to review the use of mHealth or eHealth technologies for the promotion of physical activity among older adults; the mHealth or eHealth interventions targeted generally healthy older people aged ≥50 years; and outcomes were related to levels of physical activity, exercise, fitness, or reduction in sedentary behavior, measured using self-report instruments, measurement devices (eg, accelerometers, pedometers), or energy expenditure (eg, metabolic equivalents of task [METs]).

Reviews were excluded if they did not use systematic review techniques, did not focus on older populations aged ≥50 years, focused on mHealth or eHealth interventions only in disease-specific samples or in people with chronic conditions (eg, Parkinson’s disease, stroke, diabetes, cancer, depression, obesity), or focused solely on technology interventions that required equipment other than body-worn sensors (eg, smartwatches) or smartphone-type devices, which are costly and unlikely to be easily accessible to older people (ie, we excluded exergaming, virtual reality gaming, smart homes, robotics).

Reviews in which only a minority of the included studies met the described criteria (ie, only some of the included studies focused on older adults, only some of the included studies focused on generally healthy populations, the majority of technologies were outside our inclusion criteria) were considered for inclusion only if it was agreed between both authors independently that they provided insight into the effectiveness of mHealth or eHealth interventions. In order to be accepted for inclusion, the review had to present analysis for the subset of relevant studies included in the review that fulfilled our inclusion criteria, or it had to be possible to derive estimates of effect for the subset of relevant studies included in the review from data presented.

### Screening and Data Extraction

Following identification and removal of duplicates, we exported all citations from Zotero (version 5.0.85) [16] into Rayyan [17], a web application to expedite the blinded screening process. Titles, abstracts, and potentially relevant full texts were screened independently by both authors, and any disagreements were resolved by discussion. Both authors independently used a tailored, predefined, data extraction form to record relevant review characteristics (Multimedia Appendix 2 for data extracted). Descriptions of the included reviews were tabulated for clarity.

### Data Analysis

Depending on heterogeneity of interventions and outcome measurements and summary measures used across included systematic reviews, we undertook a meta-analysis [18]. If the meta-analysis was not possible, we present pooled summary data without further analysis.

### Quality Assessment

Included reviews were subjected to quality assessment using AMSTAR-2 [19,20]. AMSTAR-2 is used to generate an overall rating of “high,” “moderate,” “low,” or “critically low.” Both authors conducted the assessments independently and then discussed ratings to agree consensus. Assessments of study quality or risk of bias reported in the included systematic reviews are presented (Table 1).

**Table 1:** Summary of review characteristics and quality assessment.

Author (year)	Type of review	Number of included studies and designs	Aim	Population (n)	AMSTAR-2 Rating	Quality of evidence^a^
Cooper et al (2018) [21]	Systematic review and meta-analysis	9 RCTs^b^ (8 included in meta-analysis)	To investigate how different wearable activity trackers impact PA^c^ levels	Older adults aged ≥65 years (939)	Moderate	Cochrane Risk of Bias Tool: high risk (n=6), unclear risk (n=2), low risk (n=1)
Jonkman et al (2018)^d^ [22]	Narrative review, but systematic approach	12 RCTs	To provide an overview of the effectiveness of eHealth interventions in increasing PA in older adults	Community-dwelling adults aged ≥55 years (1208)	Critically low	Not assessed
Larsen et al (2019) [23]	Systematic review and meta-analysis	21 studies: 20 RCTs, 1 RCT with crossover design	To estimate the effect of PAM^e^-based interventions on PA behavior	Adults aged ≥65 years (2783)	High	Cochrane Risk of Bias Tool (v2.0): high risk (n=6), moderate risk (n=10), low risk (n=5)
Muellmann et al (2018) [24]	Systematic review	20 studies: 18 RCTs, 2 quasiexperimental designs	To compare the effectiveness of eHealth interventions promoting PA in older adults	Adults aged ≥55 years (6671)	Moderate	Cochrane Risk of Bias Tool: high risk (n=8), moderate risk (n=11), low risk (n=1)
Yerrakalva et al (2019) [25]	Systematic review and meta-analysis	6 studies (5 included in meta-analysis): 5 RCTs, 1 NRSI^f^	To quantify the effect of mHealth^g^ app interventions on sedentary time, PA, and fitness	Community-dwelling adults aged ≥55 years (486)	High	GRADE^h^ assessment: moderate-certainty evidence for PA and sedentary time; low-certainty evidence for fitness

^a^Quality of the evidence as assessed by the systematic review authors.

^b^RCTs: randomized controlled trials.

^c^PA: physical activity.

^d^Excluded from this overview based on AMSTAR assessment.

^e^PAM: physical activity monitor.

^f^NRSI: nonrandomized study of interventions.

^g^mHealth: mobile health.

^h^GRADE: Grading of Recommendations, Assessment, Development and Evaluations.

## Results

**Figure 1:**
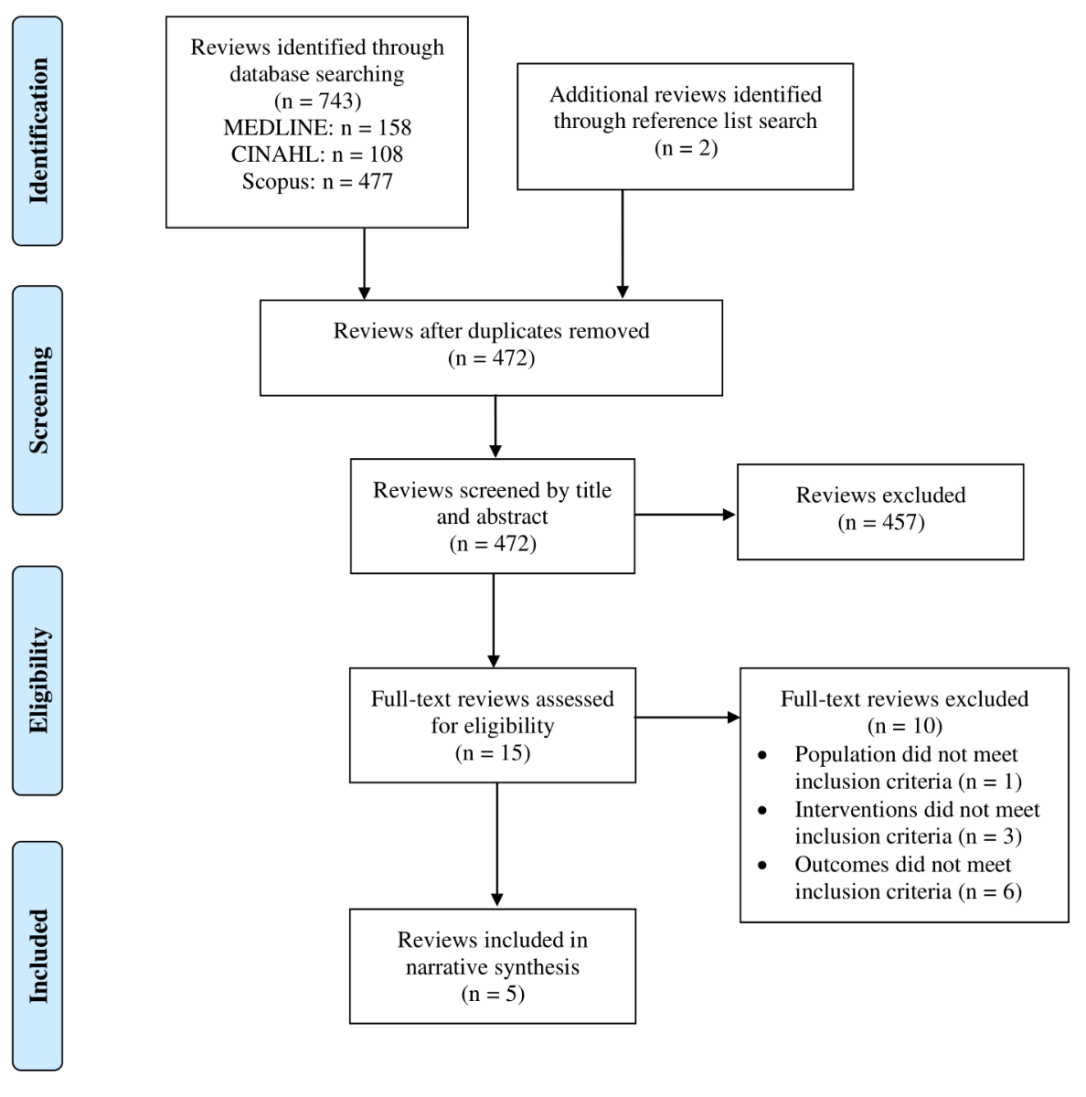
PRISMA flow diagram of the inclusion process.

The search process is illustrated in Figure 1. We identified 472 potentially relevant articles, after removal of duplicates. Following title and abstract independent screening, 15 reviews remained for full-text screening by both reviewers. Reasons for exclusion are listed in Multimedia Appendix 3. Overall, 5 reviews were selected for inclusion. Of these, 3 were systematic reviews with meta-analyses that provided pooled estimates of the effect of the technologies reviewed, 1 was a systematic review with a narrative synthesis, and 1 was a narrative review that used systematic review methods (Cochrane guidelines) for search selection and data extraction. Study characteristics and quality assessments for each included review are presented in Table 1. There was 100% agreement on AMSTAR-2 quality ratings without need for consensus discussions. Two of the reviews were rated as high quality and 2 as moderate quality. The quality of 1 review [22] was assessed as critically low, meaning that “the review should not be relied upon to provide an accurate and comprehensive summary of the available evidence” [19]. The review was not strictly a systematic review, although it followed a systematic approach, and this may have contributed to the poor AMSTAR-2 rating. This review is not discussed further. For completeness, details of this review are presented in Tables 1 and 2.

Because of the heterogeneity of methods, interventions, and outcomes, we were unable to undertake a meta-analysis of the reviews included.

**Table 2:** Summary of review interventions, comparators, outcomes, and conclusion regarding intervention effectiveness.

Author (year)	mHealth^a^ or eHealth intervention	Comparator or control	Key outcomes	Effectiveness of intervention
Cooper et al (2018) [21]	Wearable motion sensing technology interventions (accelerometer or pedometer) designed to increase PA^b^; duration range ≥6 weeks, ≤52 weeks	Usual care or standard care, waitlist control, or other active comparator focused on enhancing PA (eg, educational or behavioral interventions)	Change in PA behavior (minutes walking per day, steps per day, proportion of participants at activity goal)	Statistically significant effect of using accelerometers (SMD^c^=0.43 [95% CI 0.19 to 0.68, I^2^=1.6%^d^]), but not pedometers (SMD=0.17 [95% CI −0.08 to 0.43, I^2^=37.7%]) for increasing PA levels
Jonkman et al (2018)^e^ [22]	Computer, tablet, smartphone, or smartwatch technology to promote PA or reduce sedentary behavior; duration range ≥4 weeks, ≤6 months	Usual practice, waitlist control, or other active comparator (eg, pedometer, blinded activity tracking)	Objective assessment of the amount of PA (eg, daily step counts, minutes spent on PA)	Positive short-term effect of increased PA (ie, right after administering the intervention), but lacking evidence for long-term effects
Larsen et al (2019) [23]	Any PAM^f^-based intervention (ie, accelerometer or pedometer) where the participants of the intervention group received any kind of feedback on their PA level measured by PAMs; duration range ≥4 weeks, ≤52 weeks	No feedback on PA level is given from the PAMs	PA (steps per day) as primary outcome; secondary outcomes included: MVPA^g^, sedentary time, physical capacity, HRQoL^h^	Statistically significant effect on PA favoring the intervention (SMD=0.54 [95% CI 0.34 to 0.73, I^2^=79.2%]); statistically significant effect on MVPA favoring the intervention (SMD=0.34 [95% CI 0.15 to 0.52, I^2^ = 65.8%]); inconclusive results for effects on sedentary time; no effect on physical capacity, BMI, or HRQoL
Muellmann et al (2018) [24]	The main intervention component delivered via computer (ie, website, PDA, virtual advisor), phone, or text messaging; duration range ≥4 weeks, ≤24 months	Non-eHealth intervention (eg, paper-pencil intervention without eHealth component, face-to-face consultation such as prescription of PA by a physician, or exercise in groups or with a personal trainer) or no intervention	PA assessed using objective measures (eg, pedometer, accelerometer), subjective measures (eg, PA diary, questionnaires), or a combination of objective and subjective methods	Overall, the 9 studies that used web-based interventions appear to have beneficial effects on increasing PA compared to various comparators (no intervention or paper-based interventions) in the short-term (1-6 months) with small effect sizes in the range of 0.20-0.31.
Yerrakalva et al (2019) [25]	mHealth app intervention delivered via smartphones or tablet computers; in 5 of 6 studies, the app synced with a wearable device (pedometers or wearable smart device); duration range ≥3 months, ≤6 months	Modified dose of intervention (modified volume of intervention or modified version of same app), different app, non-app intervention, or no intervention	PA (active minutes per day; steps per day), physical fitness (maximal oxygen uptake, 6-minute timed walk, gait speed), and sedentary time (% sedentary time per day, sitting time per day)	Interventions may be associated with increased PA (pooled mean difference 506 steps/day [95% CI −80 to 1092, I^2^=80.5%]), decreased sedentary time (SMD=−0.49 [95% CI −1.02 to 0.03, I^2^=0%]), and increased fitness (SMD=0.31 [95% CI −0.09 to 0.70, I^2^=0%]) in trials ≤3 months and with increased PA (753 steps/day) in trials ≥6 months. Results for all individual outcomes revealed trends in the same direction, but all results were inconclusive as the CIs included zero.

^a^mHealth: mobile health.

^b^PA: physical activity.

^c^SMD: standardized mean difference.

^d^Measure of heterogeneity.

^e^Excluded from this overview based on AMSTAR assessment.

^f^PAM: physical activity monitor.

^g^MVPA: moderate-to-vigorous physical activity.

^h^HRQoL: health-related quality of life.

### Types of mHealth and eHealth Interventions

**Figure 2:**
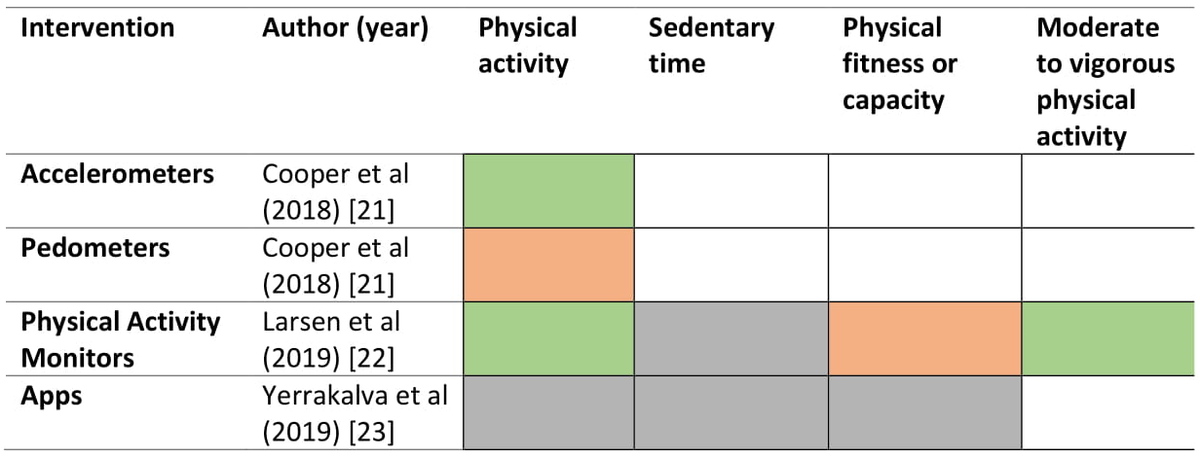
Summary of evidence from meta-analyses. Green indicates the intervention was effective, amber indicates there was no difference in the investigated comparison, grey indicates the evidence was inconclusive, and no color (blank) indicates the outcome was not assessed.

An overview of the interventions, comparators, outcomes, and effectiveness of the interventions described in the reviews are presented in Table 2. In 2 reviews, interventions were delivered via wearable motion sensing devices (eg, accelerometers or pedometers) [21,23]. In 1 review, interventions were delivered via apps on smartphones or tablets, and all but one of the included studies involved syncing the app to wearable devices [25]. One review [24] included a mix of interventions, including computer-based (ie, websites, personal digital assistant, virtual advisor; n=9), phone (n=7), and text messaging (n=4), with a separate narrative synthesis provided for each mode of intervention. The overall intervention duration for studies included in reviews ranged from 4 weeks to 2 years. All reviews reported on physical activity as a key outcome. Figure 2 presents a summary of evidence from meta-analyses for each intervention.

### Reviews Reporting Significant Effects of Interventions

Significant effects of mHealth or eHealth interventions on physical activity outcomes were reported by 3 reviews.

Larsen et al [23] reviewed 21 studies on the effects of physical activity monitors (PAM) on physical activity, sedentariness, physical capacity, BMI, and self-reported health-related quality of life (HRQoL) compared to control interventions. Of these studies, 20 were RCTs with parallel group design, and 1 was a crossover RCT. Although 6 studies included samples with specific diagnoses, subgroup analysis revealed no significant differences across any of the outcomes in relation to diagnoses. Regarding the risk of bias, 5 studies were rated as having low risk of bias, 10 studies as medium risk, and 6 studies as high risk of bias. Results of a random effects meta-analysis on the effect of PAM intervention on physical activity found a moderate standardized mean difference (SMD) of 0.54 (95% CI 0.34 to 0.73) in support of the PAM interventions, equivalent to about 1300 more steps per day. Furthermore, there was a small to moderate effect on moderate-to-vigorous physical activity equivalent to 8 more minutes activity per day (SMD 0.35, 95% CI 0.15 to 0.52), favoring intervention. Heterogeneity was high for both comparisons. The pooled effects for time spent sedentary, physical capacity, BMI, and self-reported HRQoL were not significant. Effects are not reported in relation to length of the intervention, but sensitivity analysis found that intervention length was not significantly correlated with the effect size for any outcomes. Overall, the quality of the evidence was judged as low to moderate due to unexplained heterogeneity, publication bias, and imprecision. However, the authors conclude that PAM-based interventions are safe and feasible for use in older adult populations.

Cooper et al [21] reviewed 9 RCTs that investigated the effect of accelerometer and pedometer use on physical activity in older adults, 8 of which were included in the meta-analysis. Most studies (n=6) were judged to be at high risk of bias, 2 were unclear, and 1 was considered to be at low risk of bias. Pooled results from 4 studies investigating the effect of accelerometers revealed small to moderate positive effects on physical activity (SMD 0.43, 95% CI 0.19 to 0.68). Pooled results from 4 studies investigating pedometers revealed no statistically significant effect on physical activity (SMD 0.22, 95% CI –0.08 to 0.51). Overall, shorter duration accelerometer interventions appeared to have a larger effect, but intervention duration and individual study estimates were variable. Adherence to the intervention was reported in 5 of the 9 included studies and was high on average (≥80%). The authors concluded that higher step detection accuracy in accelerometers may explain why feedback from accelerometers was found to increase levels of physical activity, whereas pedometers did not, but caution that the high risk of bias found in most studies limits conclusions that can be drawn from these findings.

Muellmann et al [24] reviewed 20 studies comparing the effects of eHealth interventions on physical activity in older people (≥55 years). Only 1 study was assessed as having low risk of bias. Heterogeneity in intervention, mode of delivery, duration, outcome assessments, and comparator groups precluded meta-analysis. There were 18 RCTs and 2 quasiexperiments. Website-based interventions were used in 9 studies. These studies overall appear to have beneficial effects on increasing physical activity compared to comparators (no intervention or paper-based interventions) in the short-term (1-6 months) with small effect sizes in the range of 0.20-0.31. Of the 7 studies using telephone-based interventions (health education, telephone fitness sessions, and advice), 3 studies reported no effect, while 4 reported effects of improved activity. Observed effects were most common over the shorter term. Of the 4 studies including text-messaging interventions, 3 reported positive effects of text messaging over periods up to 3 months but not beyond. The authors conclude that “eHealth interventions can effectively promote PA in older adults aged 55 years and above when compared to no intervention control groups at least in the short term.” Interventions that are theory-based were more effective regardless of intervention mode, and greater engagement was associated with effect, but participants seldom reached intended exercise dose. The authors urge caution, as the risk of bias in the studies reviewed was high to moderate and there was great heterogeneity in intervention mode, content, and duration.

### Reviews Reporting Inconclusive Effects of Interventions

One of the reviews, by Yerrakalva et al [25], found no significant effects of app-based interventions on physical activity outcomes. The review included 6 studies (5 RCTs); studies appear underpowered, with all but 1 having total sample sizes <65 and intervention groups <25 participants. For the RCTs, risk of bias was judged to be low. The pooled analyses reported the following: for physical activity, average increases of 506 steps per day over 3 months and 753 steps per day over 6-12 months; for sedentary behavior, reductions in sedentary time at 3 months and 6 months; for physical fitness, small increases in fitness up to 3 months expressed in a number of different ways (gait speed, timed walk, VO_2_ max). Results reveal trends in the same direction for an effect of the apps but have to be judged as inconclusive as confidence intervals included zero. Features that appeared to be common to apps demonstrating improvement trends included syncing to smartwatches or activity monitors and behavior change techniques including goal setting, self-monitoring, instructions for use, social rewards, and combining the app with professional support.

## Discussion

We undertook a rapid review of systematic reviews to assess the evidence for mHealth or eHealth interventions in the promotion of physical activity among older people. We initially included 5 reviews but excluded 1 (critically low quality) from the overview. We were unable to undertake meta-analysis of the reviews because of the heterogeneity of the methods, interventions, and outcomes. Three reviews [21,23,24], 2 of which included meta-analyses, found that eHealth approaches (activity monitors, web-based interventions) improved physical activity outcomes. One review and meta-analysis [25] reported trends toward an intervention effect; however, results were inconclusive. Although risk of bias was judged to be low in the studies included in the review, they appeared underpowered, with small total and group sample sizes, potentially explaining why no significant effects were found. Overall, there is evidence from 3 moderate- to high-quality systematic reviews to support the effectiveness of mHealth or eHealth approaches in increasing physical activity, at least over the short term. Due to reported risk of bias in the studies included in the reviews, the overall quality of this evidence is judged as low to moderate.

The reviews identified several components of successful interventions that align with behavior change techniques known to increase motivation and facilitate behavior modification [26]. Most of the studies involved elements of self-monitoring and feedback, either in the form of apps or wearable devices. The provision of real-time feedback on individuals’ levels of physical activity was associated with significant increases in the behavior [24], and the implementation of motivational tools such as self-monitoring and feedback as a means of positively impacting levels of physical activity, goal attainment, and adherence has previously demonstrated success [21]. Interventions that were theory-based were more effective, and effect was associated with greater engagement regardless of the mode of intervention [24]. Although Yerrakalva et al [25] determined the effect of the intervention was inconclusive, they reported on the common features of the studies that found app interventions to be effective. These included self-monitoring, goal setting, clear instruction on how to perform the behavior, and social and professional support. Support from professionals may be particularly important in encouraging engagement and adherence to home exercise. A review on home-based, nontechnology physical activity interventions in older adults [27] found that contacting individuals by phone in order to provide support was a good alternative to onsite supervision. They reported strong evidence, based on 3 studies, indicating that direct remote contact had positive effects on physical activity and capacity measures, to a similar extent as supervised training.

An important consideration for mHealth or eHealth interventions concerns the practicalities of implementing technological solutions, especially when users have limited experience of using such devices [28]. This is an important point to keep in mind for the older and disadvantaged sectors of the population, whom we already know are likely to have less experience of technologies [29]. Consideration must be given to the “digital divide” that may result from socioeconomic status, age, geographic location, and cultural factors [28]. Technologies need to provide activity interventions that fit in with older peoples’ lifestyles and expectations and offer tailored interventions taking account of individual preferences and capabilities. A review of older adults’ perceptions of technologies [30] found that one size does not fit all and technologies need to be tailored to individual need. Intrinsic factors related to older adults’ attitudes towards technologies centered around control, independence, and perceived need or requirement for safety are all important for motivation to use the technologies. Extrinsic factors identified were related to usability, feedback gained from technology, and costs. If older people are to use technologies, the positive benefits need to be emphasized and clearly recognizable, including how the technology promotes independence. Technologies need to be perceived as reliable and effective if they are to engender long-term use. Acceptability of technology and adherence to the interventions were not key outcomes in any of the reviews included in our review. Even so, adherence was high on average in 5 of the studies included in 1 review [21]. Wearable devices such as accelerometers and pedometers are feasible for use with older adult populations, with devices attached to the wrist or ankle being most commonly accepted in older groups [31].

This rapid review of reviews used rigorous systematic methods in the search of the literature and the assessment of review quality, and we excluded reviews of low methodological quality. Nevertheless, reviews of reviews can be limited as they face the challenge of synthesizing information in the presence of inevitable heterogeneity. This had a clear effect in this review resulting in our inability to meta-analyze results from previous reviews. Even so, this type of review allows a greater scope for generality in research findings and provides an accessible summary of evidence to support decision making by health care professionals or policy makers [14].

In conclusion, the use of mHealth or eHealth interventions with older people may be effective in increasing physical activity and physical fitness and decreasing sedentary time, over the short term. However, the evidence is currently not conclusive. These findings are in line with those relating to mHealth interventions to increase activity and reduce sedentary behaviors for all age groups [10]. Furthermore, our review suggests that interventions that are theory-based and include behavior change techniques, clear instructions, and social and professional support may be more effective than those that do not. When introducing new technologies such as apps to older people, the steep learning curve older people may experience must be recognized and support supplied to help them become familiar with the technology. Given the ongoing crisis caused by COVID-19 and the challenges we face as we reorganize services in a post-COVID-19 world, there is clearly great potential for digitalization of services for older people, although differences in uptake could exacerbate health inequalities if access is not made available to all groups. The evidence for the effectiveness of mHealth or eHealth provision of interventions to support and promote physical activity among older people is still in its infancy, but nonetheless promising. Future research requires high-quality RCTs comparing different modes of delivery, but implementation may require faster turnaround and should therefore be rigorously evaluated.

## Appendix

Multimedia Appendix 1 Search terms.

Multimedia Appendix 2 Data extracted.

Multimedia Appendix 3 Excluded full text articles with reasons.

## Abbreviations

HRQoL: health-related quality of life
METs: metabolic equivalents of task
PAM: physical activity monitor
PRISMA: Preferred Reporting Items for Systematic Reviews and Meta-Analyses
RCT: randomized controlled trial
SMD: standardized mean difference
