# Supplementary material for: Promotion of Physical Activity in Older People Using mHealth and eHealth Technologies: Rapid Review of Reviews
Source: J Med Internet Res. 2020 Dec 29;22(12):e22201. doi: 10.2196/22201 (PMC7803474; doi:10.2196/22201)
Supplement: Multimedia Appendix 1 [file jmir_v22i12e22201_app1.pdf]

Database	Search Terms	Output
MEDLINE (Ovid)	1. Older adults.mp.<br>2. Older people.mp.<br>3. elderly.mp.<br>4. mhealth.mp.<br>5. ehealth.mp.<br>6. telehealth.mp.<br>7. Mobile Applications/ or mobile technology.mp.<br>8. technolog*.mp.<br>9. smartphones.mp.<br>10. mobile health.mp.<br>11. physical activity.mp. or Exercise/<br>12. falls.mp. or Accidental Falls/<br>13. falls prevention.mp.<br>14. adherence.mp. or "Treatment Adherence and Compliance"/<br>15. Health* Behavio?r/ or Health Promotion/ or promotion.mp.<br>16. Behavio?r change.mp.<br>17. "Review"/ or "Systematic Review"/<br>18. 1 or 2 or 3<br>19. 4 or 5 or 6 or 7 or 8 or 9 or 10<br>20. 11 or 12 or 13 or 14 or 15 or 16<br>21. 17 and 18 and 19 and 20<br>22. cancer.ti.<br>23. diabet*.ti.<br>24. obesity.ti.<br>25. depress*.ti.<br>26. 22 or 23 or 24 or 25<br>27. 21 not 26	158
Scopus (Elsevier)	TITLE-ABS-KEY (("older adults" OR elderly OR "older people") AND (mhealth OR ehealth OR technol* OR telehealth OR "mobile application*" OR smartphone OR "mobile health") AND ("physical	477

	activity" OR exercise OR fall* OR "fall* prevention" OR adherence OR "health behav*" OR "health promotion" OR "behav* change") AND (review OR "systematic review") AND NOT (cancer OR obesity OR depress* OR diabet*))	
CINAHL Plus (EBSCO)	AB ("older adults" or "older people" or elderly) AND AB (mhealth or m-health or "mobile health" or "health app" or "health applications" or ehealth or e-health or telehealth or smartphone* or technolog*) AND AB ("physical activity" or exercise or fall* or "fall* prevention" or adherence or "health* behavio#r" or "health promotion" or promotion or "behavio#r change") AND AB (review or "systematic review") NOT TI (cancer or diabet* or obesity or depress*)	108
