# Supplementary material for: Promotion of Physical Activity in Older People Using mHealth and eHealth Technologies: Rapid Review of Reviews
Source: J Med Internet Res. 2020 Dec 29;22(12):e22201. doi: 10.2196/22201 (PMC7803474; doi:10.2196/22201)
Supplement: Multimedia Appendix 2 [file jmir_v22i12e22201_app2.pdf]

Data extracted from the reviews included: author and year of publication; design; number of included studies and their designs; aim of review; description of included population/s; description of mHealth/eHealth intervention/s; comparators; key outcome/s; effectiveness of interventions; and an overall statement on quality appraisal.
