# Supplementary material for: Promotion of Physical Activity in Older People Using mHealth and eHealth Technologies: Rapid Review of Reviews
Source: J Med Internet Res. 2020 Dec 29;22(12):e22201. doi: 10.2196/22201 (PMC7803474; doi:10.2196/22201)
Supplement: Multimedia Appendix 3 [file jmir_v22i12e22201_app3.pdf]

Reviews excluded	Reason(s) for exclusion
Aalbers, T., Baars, M. A. E., & Rikkert, M. O. (2011). Characteristics of effective Internet-mediated interventions to change lifestyle in people aged 50 and older: a systematic review. Ageing research reviews, 10(4), 487-497.	Outcomes did not meet inclusion criteria – The majority of studies included (10 out of 12) focus on outcomes other than physical activity (e.g. nutrition and weight-loss). Two papers with physical activity outcomes were not analysed independently.
Alharbi, M., Straiton, N., Smith, S., Neubeck, L., & Gallagher, R. (2019). Data management and wearables in older adults: A systematic review. Maturitas, 124, 100-110.	Outcomes did not meet inclusion criteria – Did not clearly address the effectiveness of wearable devices on physical activity outcomes in older people.
Bert, F., Giacometti, M., Gualano, M. R., & Siliquini, R. (2014). Smartphones and health promotion: a review of the evidence. Journal of medical systems, 38(1), 9995.	Outcomes did not meet inclusion criteria - Six of the studies in the review relate to health in the elderly but the outcomes are not physical activity.
De Bruin, E. D., Hartmann, A., Uebelhart, D., Murer, K., & Zijlstra, W. (2008). Wearable systems for monitoring mobility-related activities in older people: a systematic review. Clinical rehabilitation, 22(10-11), 878-895.	Outcomes did not meet inclusion criteria - Did not clearly address the effectiveness of the technology on physical activity in older people. Focuses more on feasibility of the technology.
Geraedts, H., Zijlstra, A., Bulstra, S. K., Stevens, M., & Zijlstra, W. (2013). Effects of remote feedback in home-based physical activity interventions for older adults: a systematic review. Patient education and counseling, 91(1), 14-24.	Intervention did not meet inclusion criteria - The home exercise interventions are not mHealth/eHealth. The focus of the review is the effect of remote feedback (via phone call).
Gordt, K., Gerhardy, T., Najafi, B., & Schwenk, M. (2018). Effects of wearable sensor-based balance and gait training on balance, gait, and functional performance in healthy and patient populations: a systematic review and meta-analysis of randomized controlled trials. Gerontology, 64(1), 74-89.	Population did not meet inclusion criteria - One of the eight included studies was in a generally healthy older population and this was not included in the meta-analysis. All analyses in this review are dependent on patient populations (e.g. stroke, Parkinson's).
Kampmeijer, R., Pavlova, M., Tambor, M., Golinowska, S., & Groot, W. (2016). The use of e-health and m-health tools in health promotion and primary prevention among older adults: a systematic literature review. BMC Health Services Research, 16(5), 290.	Outcomes did not meet inclusion criteria – The study focused on the scope of use of mHealth and eHealth tools, with an emphasis on barriers and facilitators.
Müller, A. M., & Khoo, S. (2014). Non-face-to-face physical activity interventions in older adults: a systematic review. International Journal of Behavioral Nutrition and Physical Activity, 11(1), 35.	Intervention did not meet inclusion criteria - No insight provided into eHealth/ mHealth as they comprised only a subset of the papers and were not analysed independently.
Taraldsen, K., Chastin, S. F., Riphagen, I. I., Vereijken, B., & Helbostad, J. L. (2012). Physical activity monitoring by use of accelerometer-based body-worn sensors in older adults: a systematic literature review of current knowledge and applications. Maturitas, 71(1), 13-19.	Outcomes did not meet inclusion criteria - Studies included focused on the ability of the technology to measure/track activity patterns. The review did not focus on effectiveness of the technology on physical activity.

Valenzuela, T., Okubo, Y., Woodbury, A., Lord, S. R., & Delbaere, K. (2018). Adherence to technology-based exercise programs in older adults: a systematic review. Journal of Geriatric Physical Therapy, 41(1), 49-61.	Intervention did not meet inclusion criteria – The primary intervention was exergaming.
